# Targeting Recipient Dendritic Cells with Sialic Acid-Modified Donor Alloantigen Prolongs Skin Transplant Survival

**DOI:** 10.3390/ijms26136168

**Published:** 2025-06-26

**Authors:** Monica Sen, Qi Peng, Kulachelvy Ratnasothy, Martino Ambrosini, Hakan Kalay, Jordan Bazoer, Kate E. Adams, Nouhad El Ouazzani, Abdessamad Ababou, David B. Guiliano, Jose I. Saldaña, Yvette van Kooyk, Giovanna Lombardi, Lesley A. Smyth

**Affiliations:** 1 School of Health, Sport and Bioscience, University of East London, Water Lane, London E15 4LZ, UK; 2 MRC Centre for Transplantation, Peter Gorer Department of Immunobiology, School of Immunology & Microbial Sciences, King’s College London, London SE1 9RT, UKgiovanna.lombardi@kcl.ac.uk (G.L.); 3 DC4U Technologies, 3621 ZA Breukelen, The Netherlands; 4 Department of Molecular Cell Biology and Immunology, Amsterdam UMC, Location Vrije Universiteit Amsterdam, O12 Building Postbox 7057, 1007 MB Amsterdam, The Netherlands; 5 School of Cardiovascular Medicine and Science, King’s College London, London SE1 1UL, UK; 6 School of Life Sciences, University of Westminster, London W1B 2HW, UK; 7 Section of Cell Biology, City St. George’s University of London, Cranmer Terrace, London SW17 0RE, UK; 8 School of Medicine and Biomedical Sciences, University of West London, St. Mary’s Road, Ealing, London W5 5RF, UK

**Keywords:** tolerance, dendritic cells, siglecs, allorecognition

## Abstract

Mature dendritic cells (DCs) are known to activate effector immune responses, whereas steady state immature DCs can induce tolerance. Several studies have targeted immature murine quiescent DCs in vivo with antigen, including donor alloantigens, for the induction of tolerance. Receptors expressed by specific DC subsets have been also targeted with antibodies linked with antigens to induce tolerance; for instance, in vivo targeting of the DCIR2^+^ DC subset with donor alloantigen resulted in long-term survival of heart and skin transplants. DCs also express sialic acid immunoglobulin-like lectin (Siglec) receptors, and these have been successfully targeted with myelin oligiodendrocyte glycoprotein (MOG) antigen to induce tolerance in experimental autoimmune encephalomyelitis (EAE). We investigated, in a mismatched model of skin transplant (B6K^d^ into B6 recipient mice), whether targeting a sialylated alloantigen K^d^ (Sia-K^d^) to Siglecs on recipient DCs promoted transplant survival. The injection of α2,3 Sia-K^d^ into B6 recipient mice prior to B6K^d^ skin transplantation, by binding to Batf3 dependent DCs, resulted in prolonged skin graft survival and an increase in CD4^+^CD62L^+^Foxp3^+^ Tregs. Targeting Siglecs on DC subsets in vivo represents a novel way of improving transplant survival.

## 1. Introduction

Dendritic cells (DCs) are key cellular players involved in the regulation of immune responses due to their ability to traffic from the periphery to the lymph nodes (LNs), to undergo maturation, and to present antigen to antigen-specific T cells. Whether the end point is the activation of the immune system or tolerance is very much dependent on the DC subset involved, their maturation status, and the microenvironment [1,2,3].

In the context of transplantation, DCs are the main cell type responsible for the recognition of alloantigen by recipient T cells. Donor DCs, present in the graft at the time of transplantation, present alloantigens directly to recipient T cells [4,5], while recipient DCs present alloantigens indirectly [6,7], as a peptide in the context of recipient MHC molecules, or as intact alloantigens directly following acquisition [8,9,10,11]. Given the key role of recipient DCs in shaping the immune response during transplantation, several strategies utilising these cells to induce transplant tolerance have been assessed. The in vitro manipulation of recipients/autologous DCs, either by genetic modification [12] or following treatment with drugs (e.g., rapamycin [13], dexamethasone [14,15], retinoic acid [16], aspirin [17], and vitamin D3 [15]) or cytokines (e.g., IL-4, IL-10, low dose GM-CSF [18,19,20]), led to the generation of DCs with ‘tolerogenic’ functions. The adoptive transfer of these ‘tolerogenic’ autologous DCs in vivo resulted in tolerance induction in animal models and improved disease outcome in the clinic [21,22]. For example, bone marrow (BM)-derived recipient DCs (BMDCs) rendered ‘tolerogenic’ in vitro have been successful in prolonging allograft survival in animal models [22,23]. Recipient-derived ‘tolerogenic’ DCs have also been injected into kidney recipient patients as a phase I clinical trial, as part of the One Study [24], and recently Moreau et al. (2023) also published the outcome of a phase I/IIa study of kidney transplant recipient treated with autologous tolerogenic cells [25]. These authors highlighted that autologous tolerogenic DCs were safe, with 100% graft survival observed during the three-year follow up period. Additionally, they observed reduced CD8^+^ T cell activation markers and increased Foxp3 expression in their DC treated patients [25]. However, adoptive cell therapy is not without its limitations, including expensive large-scale DC production and the use of standard immunosuppression, so alternative methods to create tolerogenic recipient DCs are attractive.

Strategies aiming to promote the tolerogenic programming of in vivo quiescent, immature, recipient DCs directly have proved successful and represent one such alternative. Specific receptors on conventional (classical) DCs (cDCs), consisting of Batf3 dependent type 1 (cDC1; CD103^+^ and CD8α^+^) and Batf3 independent/IRF4 dependent type 2 (cDC2; CD11b^+^) cDCs, monocyte-derived DCs (moDCs), plasmacytoid DCs (pDCs), dermal and Langerhans cells (LCs), have all been targeted with antigen to induce tolerance [26]. In a solid organ setting, we observed that targeting an alloantigen, either an MHC class I peptide or monomer, to DC immunoreceptor (DCIR2^+^) expressing CD8α^−^cDC2 using an anti-33D1 antibody led to the depletion of alloantigen-specific CD4^+^ T cells and reduced levels of IgG alloreactive antibodies [27]. Although this tolerance approach was successful, leading to prolonged skin transplant survival, in the absence of CD8^+^ T cells, it was limited as the mechanism of action appeared to be solely deletional. Previous studies have also highlighted that in vivo targeting of different DC subsets in their steady state can lead to the expansion of natural Foxp3^+^ T cells, as was the case with CD8α^−^DCIR2^+^ DCs, or the induction of Foxp3^+^ T cells by CD8α^+^DEC205^+^ DCs [28]. Therefore, targeting alloantigen to a set of receptors expressed on several DC subsets may favour transplant survival by ensuring a plethora of tolerance mechanisms.

Human and mouse immune cells, including DCs, express Siglecs [29,30]. These inhibitory receptors are type 1 transmembrane proteins, which consist of an extracellular N-terminal V-set immunoglobulin domain designated to bind α2,3, α2,6 and α2,8 linked sialic acids [30,31], with binding affinity varying between Siglecs. Siglecs E and F, present on DCs and macrophages [32], have specificity for α2,3 and/or α2,6 sialic acids, whereas α2,6-linked sialic acids are mostly recognised by Siglec CD22 expressed by B cells [33]. Recently, Borges et al. (2025) highlighted the importance of Siglecs in a transplant setting, reporting enhanced allograft rejection in Siglec E deficient mice [34]. These authors showed accelerated T cell-mediated cardiac allograft rejection in Siglec E-deficient mice through enhanced innate cell activation. This paper also highlighted that the expression of the inhibitory Siglec E receptor on DCs controlled their responses to DAMPS and limited their ability to activate alloreactive T cell responses in vitro. These authors also showed that the human homology Siglec 9 also played a similar role [34].

The antibody-mediated targeting of antigen (Ovalbumin (OVA) or myelin oligiodendrocyte glycoprotein (MOG)) to pDCs via Siglec H [35] or the uptake of α2,3-, α2,6-, or α2,8-linked sialyl-lactose (Sia) antigen (OVA or MOG) by different Siglec receptors, such as Siglec E on BM derived moDCs and cDCs, created a ’tolerogenic’ DC phenotype [36,37] capable of dampening effector T cell responses, with or without increasing the frequency of Foxp3^+^ regulatory T cells. Here, we extended this strategy to determine whether targeting Siglecs present on multiple subsets of DCs with a Sia alloantigen leads to prolonged skin transplant survival.

## 2. Results

### 2.1. Sialyated Alloantigen Binds Siglecs Expressed on DC Subsets, Leading to a Tolerogenic Phenotype

Two sialylated alloantigen peptides, α2,3 Sia-K^d^_54-68_ and α2,6 Sia-K^d^_54-68_ were constructed to assess whether targeting Siglec receptors in vivo prolonged K^d^ skin transplant survival in B6 recipients. Initial experiments were set up to assess the in vitro tolerogenic capacity of these constructs using BMDCs and/or SPLN-DCs. Siglecs E, F, G, and H as well as CD169 expression [Supplementary Figure S1a,b] were observed in the aforementioned DC populations, as was the binding of Sia-K^d^ peptides to B6 BMDCs [Supplementary Figure S2a] and α2,3 Sia-K^d^ to SPLN-DCs [Supplementary Figure S2b].

**Figure 1:**
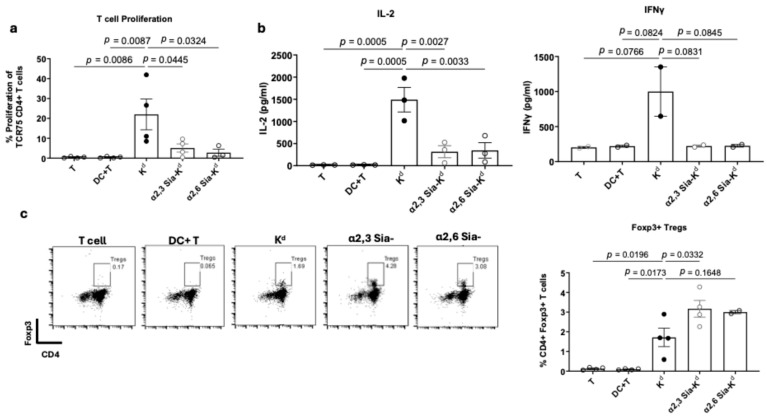
Targeting BM-DC Siglecs with Sia-K^d^ led to impaired TCR75 CD4^+^ T activation and Foxp3^+^ Tregs induced/expanded in vitro. B6 BMDCs were pulsed with 10 µg/mL of sialyated or non-sialyated peptide and co-cultured with CFSE-labelled TCR75 CD4^+^ T cells at a 1:10 ratio. After 3 days, supernatants were collected for cytokine analysis, and cells were surface stained with anti-CD4 antibody, followed by analysis of CFSE dilution. (**a**) Cells were gated via FSC and SSC, doublets were excluded, and CD4^+^ T cell proliferation was measured by CFSE dilution. Data shown is representative of four independent experiments. Bars represent mean percentages ± SEM. (**b**) IL-2 and IFNγ cytokine present in the 3-day culture supernatants were determined using an IL-2 and IFNγ sandwich ELISA, respectively. Data is representative of three independent experiments, with each bar representing the mean IL-2 or IFNγ concentration (pg/mL) ± SEM. (**c**) B6 BM-DCs were pulsed with 10 µg/mL of sialyated or non-sialyated peptide and co-cultured with TCR75 CD4^+^ T cells at a 1:10 ratio with subsequent addition of 5U IL-2 at day 0. Controls included T cells cultured alone, and T cells cultured with unpulsed DCs. After 3 days, Foxp3 expression in CD4^+^ T cells was measured following intracellular staining and subsequent flow cytometry. Cells were gated on live cells (FSC vs. SSC), and doublets were excluded followed by gating on CD4^+^ versus Foxp3^+^. Each bar represents the percentage of Foxp3^+^ Tregs from four independent experiments for all conditions except α2,6 Sia-K^d^ where the data represents two independent experiments. Data expressed as mean ± SEM with each experiment mean represented as an individual point. Statistical comparisons performed using one-way ANOVA and Tukey’s multiple comparisons test.

Antigen-specific effector T cell responses were impaired in the presence of the siaylated peptides. K^d^-specific CD4^+^ T cells, isolated from the TCR75 Rag^−/−^ transgenic mice (TCR75 T cells) [38], co-cultured in the presence of BMDCs treated with either 10 μg/mL of α2,3 or α2,6 Sia-K^d^ had significantly impaired proliferation compared to the same T cells co-cultured with K^d^ peptide pulsed DCs [Figure 1a]. In addition, and complementary to the T cell proliferation, reduced IL-2 and IFN-γ production was also observed [Figure 1b]. Like our previously published data, albeit using Sia-OVA pulsed DCs and OVA-specific T cells [36], a significant increase in CD4^+^ Foxp3^+^ Tregs was observed in the presence of BMDCs pulsed with α2,3 Sia-K^d^ peptides compared to K^d^ peptide [Figure 1c]. Taken together, we concluded that the siaylated allopeptides induced a ‘tolerogenic’ phenotype in BMDCs in vitro.

### 2.2. Skin Allograft Rejection Is Impaired Following Targeting Siglecs on Recipient DCs with α2,3 Sia-K^d^

**Figure 2:**
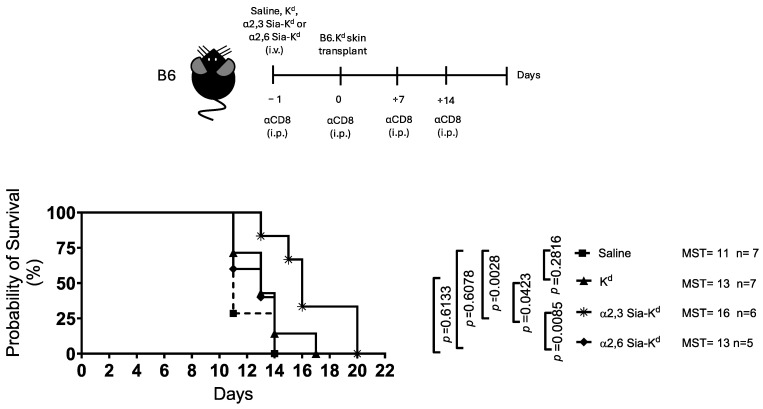
Skin transplant survival is prolonged in B6 recipient mice targeted with α2,3 Sia-K^d^ peptide. B6 mice were injected intravenously (iv) with either α2,3 Sia-K^d^ (10 μg/200 μL saline), α2,6 Sia-K^d^ (10 μg/200 μL saline), or K^d^ (10 μg/200 μL saline). Control mice received 200 μL saline only. One day later, the mice received a B6.K^d^ skin transplant (day 0). The mice received 250 μg of anti-CD8 antibody (clone YTS169) on days −1 and 0 and weekly thereafter. Skin survival was monitored daily. Experimental design (top panel). A survival graph of skin allografts (days) is shown in the bottom panel. The mean survival time (MST) for 5–7 mice groups from two independent experiments is shown. N= number of mice per group. Statistics were calculated using a log-rank (Mantel-Cox) test.

Next, we tested the effect of targeting Siglecs, with α2,3 or α2,6 Sia-K^d^, in vivo using an MHC Class 1 mismatched skin transplant model. To focus on the regulation of the indirect T cell response, mice were treated with an anti-CD8 antibody to deplete CD8^+^ T cells with direct allospecificity [27]. Recipient B6 mice received either K^d^ peptide, α2,3 Sia-K^d^, or α2,6 Sia-K^d^ complex one day before being transplanted with a full-thickness tail skin from B6 mice expressing the K^d^ transgene (B6.K^d^). We observed that α2,3 Sia-K^d^ administration significantly prolonged B6.K^d^ skin graft survival (MST: 16 days, *p* = 0.0028) compared to α2,6 Sia-K^d^ (MST: 13 days), K^d^ (MST: 13 days), or saline-treated (MST: 11 days) recipient B6 mice [Figure 2].

**Figure 3:**
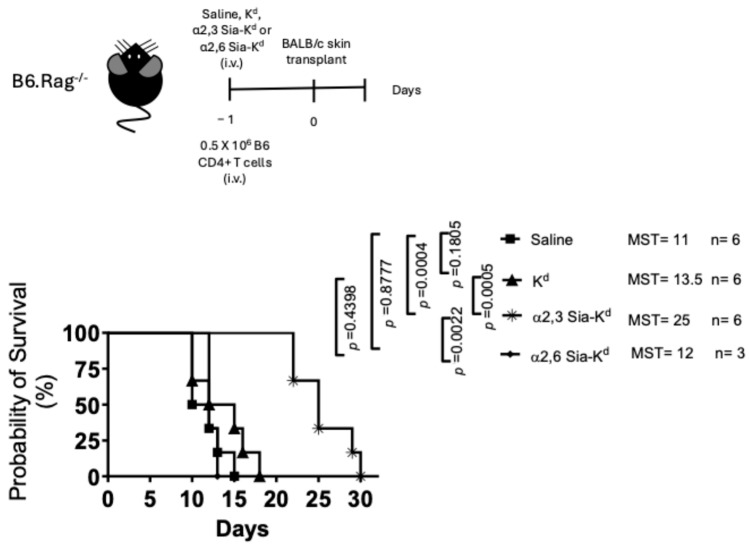
Allogeneic graft survival was prolonged in B6.Rag 2^−/−^ mice following α2,3 Sia-K^d^ treatment. Diagrammatic representation of the targeting and transplant strategy (top panel). B6.Rag 2^−/−^ mice (3–6 mice/group) received 0.5 × 10^6^ B6 CD4^+^ T cells (i.v.) and either K^d^ (10 µg/200 µL saline), α2,3 Sia-K^d^ (10 µg/200 µL saline) or α2,6 Sia-K^d^ (10 μg/200 μL saline) i.v. Control mice received 200 µL saline i.v. only. One day following peptide treatment, the mice received BALB/c skin transplant. Data are shown as percentage of mice with surviving grafts (days), and MST is shown. n = number of mice per group. Statistics were calculated using a log-rank (Mantel–Cox) test.

We next determined whether targeting Sia-K^d^ alloantigen to endogenous DCs contributed to the skin graft survival observed. Recipient B6.Rag 2^−/−^ mice that lack B and T cells, but not DCs [39], received either K^d^ peptide, α2,6 Sia-K^d^ or α2,3 Sia-K^d^ complex in conjunction with B6 CD4^+^ T cells. A BALB/c skin transplant was given one day later. In comparison to saline-treated control mice (MST: 11 days), both α2,3 Sia-K^d^ (MST: 25, *p* = 0.0005) and K^d^ peptide (MST: 13.5, *p* = 0.1805) treatments prolonged a fully mismatched skin graft survival, with the α2,3 Sia-K^d^ peptide showing the greatest efficacy [Figure 3]. This data suggests that targeting Siglecs expressed on DC in a quiescent state contributes to the reduced indirect CD4^+^ T cell-mediated skin graft rejection, as seen in our MHC I mismatched model.

### 2.3. Engaging Siglecs Expressed by Batf3-Dependent DCs with α2,3 Sia-K^d^ Prolonged Allograft Survival

**Figure 4:**
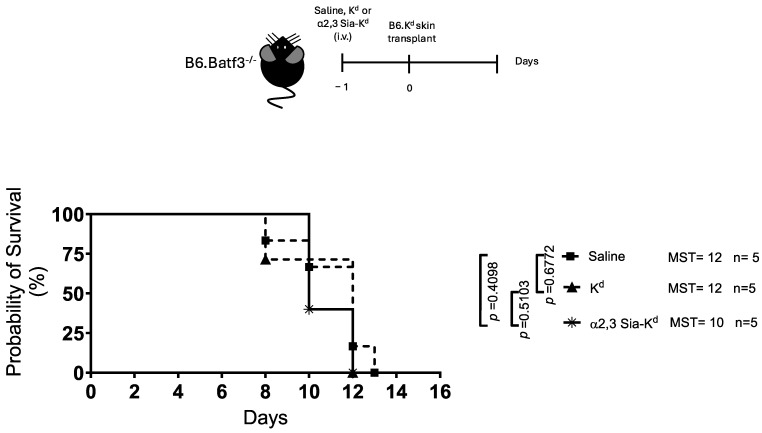
Targeting Siglecs expressed on Batf3 independent DCs with α2,3 Sia-K^d^ does not prolong allograft survival. B6.Batf3^−/−^ mice (n = 5 per group) received either α2,3 Sia-K^d^ or K^d^ (10 μg/200 μL saline) iv; control mice received 200 μL saline. One day following peptide treatment, the mice received a B6.K^d^ skin transplant. Skin survival was monitored daily. Experimental design (top panel). A survival graph of skin allografts (days) is shown in the bottom panel. MST for 5 mice per group from one independent experiment is shown. Statistics were calculated using a log-rank (Mantel–Cox) test.

We, and others, have previously reported that endogenous DCs express Siglecs. Siglecs E and F are expressed by splenic cDC1 and cDC2 [36,40,41], with Siglec G and Siglec H being reported on CD8α^+^ DCs [42] and pDCs [35], respectively. Therefore, to determine the contribution of the different Siglec-expressing DC subsets in the prolongation of skin graft survival, B6.Batf3^−/−^ mice were used as B6.K^d^ skin transplant recipients. Like our previous study, B6.K^d^ skin transplanted onto B6.Batf3^−/−^ mice was rejected with the same kinetics as a B6.K^d^ skin transplanted onto B6 mice treated with anti-CD8 antibody (rejection times 12 and 11 days for B6.Baft3^−/−^ and B6 recipient mice, respectively), suggesting that alloreactive CD8^+^ T cells in these mice do not contribute to rejection [15]. In contrast to the B6 recipient mice, no evidence of B6.K^d^ skin graft survival following α2,3 Sia-K^d^ treatment was observed in B6.Batf3^−/−^ recipient mice [Figure 4]. This result suggests that the subtype of DCs, to which α2,3 Sia-K^d^ binds to in vivo, may affect skin transplant outcome as B6.Batf3^−/−^ mice lack cDC1 DCs, both the CD8α^+^ and the CD103^+^ subsets, but they possess the cDC2s and pDCs in their lymphoid tissue [43].

**Figure 5:**
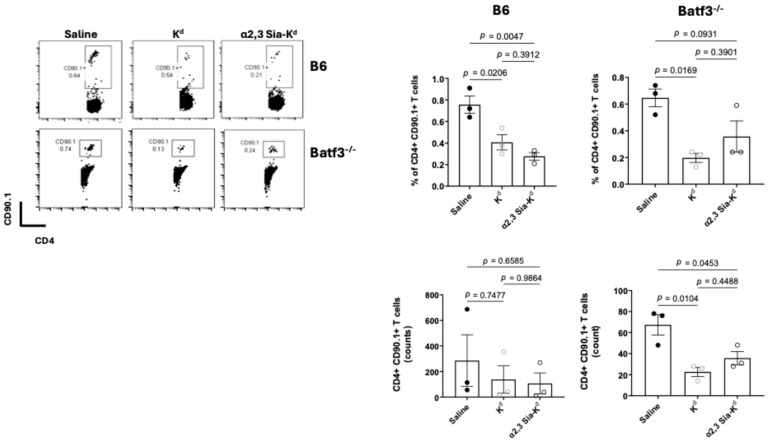
Targeting Siglec with α2,3 Sia- K^d^ leads to reduced alloreactive CD4^+^ T cells. A total of 2 × 10^6^ TCR75 T cells (CD90.1^+^) were adoptively transferred to B6 or B6.Batf3^−/−^ mice (n = 3 mice per group, data from three independent experiments) one day prior to the iv administration of 10 μg of either K^d^ or α2,3 Sia-K^d^. Controls received saline. Ten days later spleens and lymph nodes were harvested and stained for CD4 and CD90.1. The dot plots panels shown are representation data; the top and lower bar charts show bar charts of pooled data. Each data point represents the percentage of CD4^+^ CD90.1^+^ T cells (top panel) or the number of CD4^+^ CD90.1^+^ T cells (lower panel) from each individual B6 and B6.Batf3^−/−^ mouse following the treatment shown. The graph indicates the mean ± SEM. Statistical comparisons performed using one-way ANOVA and Tukey’s multiple comparisons test.

To investigate this further, TCR75 CD4^+^ T cells (CD90.1^+^) were adoptively transferred to B6.Batf3^−/−^ and B6 control mice 24 h prior to administration of either α2,3 Sia-K^d^ or K^d^ peptide, and the presence of CD4^+^ CD90.1^+^ cells was analysed 10 days later. Control mice received saline only. The percentage and number of CD4^+^ CD90.1^+^ cells were reduced in both strains of mice following K^d^ peptide or α2,3 Sia-K^d^ complex treatment, compared to controls [Figure 5], suggesting that the recognition of this antigen by TCR75 T cells presented by DCs, including the cDC2, or pDCs, present in the B6.Batf3^−/−^ mice in vivo led to either T cell depletion, impaired proliferation, or enhanced apoptosis.

The observations so far suggest that Siglecs E and F, expressed by Batf3-dependent DCs, may promote transplant survival following the α2,3 Sia targeting regime.

### 2.4. Reduced Indirect CD4^+^ T Cell Responses and Treg Expansion Following In Vitro Activation with α2,3 Sia-K^d^ Pulsed Batf3-Dependent CD103 DCs

Next, to understand the possible role of Batf3-dependent DCs, we expanded BM progenitors in the presence of FLT3L, with GMCSF to induce CD103^+^ DCs (iCD103 DCs) [44] or without GMCSF to isolate sufficient CD8α^+^ [45] for our analysis. Firstly, we confirmed Siglec expression by these cells. In contrast to the CD8α^+^ DCs only the iCD103^+^ DCs expressed Siglecs E, F, H, and CD169 [Supplementary Figure S3a]. iCD103^+^ DCs also acquired the FAM5/6-conjugated α2,3 Sia-K^d^ [Supplementary Figure S3b]. Given these results, supported further by skin transplant survival obtained with the use of anti-CD8 antibody, which depletes the CD8α^+^ DCs, only the iCD103^+^ DCs were tested in functional assays.

**Figure 6:**
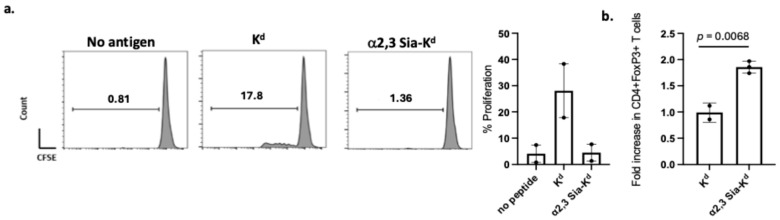
Targeting Siglecs expressed on iCD103 BMDCs with α2,3 Sia-K^d^ leads to Foxp3^+^ Tregs induced/expanded in vitro. B6 iCD103 BMDCs were pulsed with 10 μg/mL of sialyated or non-sialyated K^d^ peptide and co-cultured with CFSE-labelled CD4^+^ TCR75 T cells at a 1:10 DC: T ratio in the absence (**a**) or presence (**b**) of 5U IL-2. Controls included T cells cultured with unpulsed DCs (no antigen). After 3 days, CFSE dilution was measured by flow cytometry in the CD4^+^ T cells, and Foxp3 expression in CD4^+^ T cells was measured following intracellular staining. Cells were gated on live cells (FSC vs. SSC), and doublets were excluded followed by gating on CD4^+^ versus Foxp3^+^. (**a**) Representative histogram data from one experiment out of two performed, whilst the bar chart shows the mean ± SEM of the pooled data. (**b**) Data shows the fold increase of CD4^+^ Foxp3^+^ T cells in comparison to the control unpulsed DC:T cultures, which were set to a value of 1. Each data point represents data from two independent experiments. The graph indicates the mean ± SEM. Statistical comparisons performed using a *t*-test.

B6 iCD103 DCs were pulsed with α2,3 Sia-K^d^ peptide and cultured with TCR75 T cells. In comparison to T cells co-cultured in the presence of K^d^ peptide pulsed DCs, a lack of TCR75 proliferation was observed following stimulation with α2,3 Sia-K^d^ pulsed iCD103 [Figure 6a]. Additionally, the percentage of CD4^+^ Foxp3^+^ Tregs was significantly increased in the presence of the α2,3 Sia-K^d^ pulsed iCD103 DCs [Figure 6b].

Taken together, the data suggests that the binding of α2,3 Sia-K^d^ to CD103 DCs expands Tregs and that this increase in Treg numbers may be responsible for the prolongation survival of skin transplant seen; however, additional experiments are required to confirm these findings and to increase the reproducibility of the data.

### 2.5. Targeting Siglecs on Batf3-Dependent DCs with α2,3 Sia-K^d^ Increased CD4^+^ CD62L^+^ Foxp3^+^ Tregs Following Transplantation

**Figure 7:**
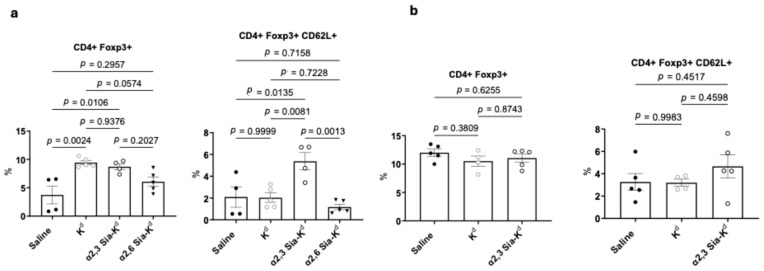
Targeting α2,3 Sia-K^d^ to Siglecs on Batf3-dependent DCs increased CD4^+^ CD62L^+^ Foxp3^+^ Tregs in transplant recipients. B6 and Batf3^−/−^ mice (n = 4–5 per group) received either K^d^ or α2,3 Sia-K^d^ or α2,6 Sia-K^d^ (10 µg/200 μL saline), iv. Control mice received saline. One day later the mice received a B6.K^d^ skin allograft. B6 mice received 250 μg of anti-CD8 antibody on days −1, +1, and +7. (**a**) B6 and (**b**) B6.Batf3^−/−^ mice were bled 14 days after transplantation, and the percentage of CD4^+^CD62L^+^Foxp3^+^ expressing Tregs was assessed by flow cytometry. CD4^+^ T cells were gated, and the percentage of Foxp3^+^ cells (left panel) and CD62L^+^Foxp3^+^ was measured. The mean ± SEM percentages of CD4^+^ Foxp3^+^ (left panel) CD4^+^CD62L^+^Foxp3^+^ Tregs (right panel) are shown; each point represents the data from an individual mouse from one independent experiment. Statistical comparison was performed using one-way ANOVA and Tukey’s multiple comparisons test.

We have previously shown that Sia-OVA treatment one week before sensitization with OVA/poly(I:C)/anti-CD40 led to an increase in the percentage of splenic CD4^+^ Foxp3^+^ T cells [36]. Here, we assess whether, following sensitization with a mismatched transplant, an increase in CD4^+^ Foxp3^+^ Tregs was observed. To this end, treated B6 mice were bled on day 14 following B6.K^d^ skin transplantation, and the percentage of CD4^+^ FoxP3^+^ Tregs measured. Given that the α2,6 Sia-K^d^ peptide did not induce skin prolongation in B6 mice, we included this construct to assess whether this correlated with the lack of Treg induction in vivo. In comparison to this construct, α2,3 Sia-K^d^ treatment increased the percentage of CD4^+^ FoxP3^+^ Tregs observed compared to untreated mice [Figure 7a, left panel, and Supplementary Figure S4]. Although the TCR75 mice are on a Rag^−/−^ background, they have detectable CD4^+^ Foxp3^+^ T cells, so the observed changes in CD4^+^ Foxp3^+^ seen may reflect the expansion of natural Tregs as well as the induction of Tregs from the CD4^+^ T cell pool. The finding that the combination of anti-CD8 antibody and K^d^ peptide treatment did not prolong graft survival but did increase the percentage of Tregs [Figure 7b, left panel] suggests that these K^d^_54–68_ peptide-specific Tregs are either not capable of suppressing CD4^+^ effector T cells that are specific for other K^d^ epitopes or do not home to the LN where alloreactive T cells reside. However, we observed that the percentage of CD4^+^CD62L^+^Foxp3^+^ cells was increased in transplanted B6 recipient mice receiving the α2,3 Sia-K^d^ construct [Figure 7b, right panel; Supplementary Figure S4]. No increase was noted following K^d^ peptide or α2,6 Sia-K^d^ treatment, as compared to untreated mice.

To confirm the requirement of Batf3-dependent DCs for Treg expansion in vivo, the experiment was repeated in B6.Batf3^−/−^ recipient mice. As expected, no increase in CD4^+^Foxp3^+^ or CD4^+^CD62L^+^Foxp3^+^ cells was observed in α2,3 Sia-K^d^-treated B6.Batf3^−/−^ transplant recipients [Figure 7b].

These results further support that the targeting of alloantigen to Siglecs expressing endogenous DCs, particularly the Batf3-dependent CD103 DCs, may be responsible for the prolongation of allograft survival.

## 3. Discussion

This study demonstrated that targeting α2,3 sialylated alloantigen to Siglec-expressing recipient DCs modified the alloresponse and reduced CD4^+^ T cell-mediated skin transplant rejection in an MHC class 1 mismatched model. Given our findings we conclude that this targeting regimen is effective at modifying recipient DCs in vivo, promoting the expansion of Tregs.

The in vitro data presented here compliments our previous study [36], suggesting that this treatment led to impaired DC function (in specific DCs subsets), very similar to what has been observed with tolerogenic DCs [15,22,46], strengthening the appeal of using sialylated alloantigen as a DC tolerance-inducing strategy. Surprisingly, our in vivo data showed that prolonged transplant survival only occurred following α2,3 and not the α2,6 Sia-K^d^ peptide administration. This may reflect the dose used; the induction of Tregs following α2,6 Sia OVA treatment has been shown to be dose-dependent [36], with different expression levels of Siglec receptors on DC subsets or cell types acquiring each construct in vivo. With respect to the latter points, we observed that BMDCs expressed more Siglec F than E whilst splenic-derived CD11c^+^ DCs have equivalent levels of both. The expression of Siglec F on CD11c^+^ SPLN-DCs has been shown histologically [41], and recently, Siglecs F and E expression on splenic cDC1 and cCD2 DCs, isolated from B6 mice, was observed by flow cytometry, albeit at a low frequency/percentage. Interestingly, the highest expression (as measured by MFI) of Siglec E was found on the cDC2s [40]. As a way of confirmation, few FLT3L expanded CD8α^+^ BMDCs (equivalent to the cDC1 cells) expressed Siglec E. Our data adds to this information, showing that the CD103^+^ iDCs, equivalent to the migratory cDC1 cells, also express high levels of Siglec F. Siglec F has been identified as a marker for the small intestine’s lamina propria (LP) CD103^+^CD11b^+^ DC lineage [47] using transcriptional profiling. However, this subset is still present in B6.Batf3^−/−^ mice, so it is not involved in the tolerance seen here. Authors of this paper also observed Siglec E at the transcriptional level in the small intestine LP CD103^+^CD11b^−^ but did not report Siglec F [47]. However, we showed that iCD103s, which are equivalent to the CD103^+^ CD11b^−^ DC lineage, do express Siglec F, suggesting that discrepancies in expression may depend on the tissue assessed. Siglec F is induced by GMCSF [48]. M-CSF-expanded BM-derived macrophages express low levels of Siglec F, which was significantly enhanced after 24 h exposure to GMCSF [48], which may explain the high expression found on GMCSF-induced BMDCs. Therefore, caution on interpreting the iCD103 data is required.

Siglec F preferentially binds α2,3 in comparison to α2,6-sialylated molecules [30,31,41]. As the internalisation of sialyated antigen leads to DCs becoming tolerogenic [36], this observation suggests that the uptake of the α2,3 constructs by Siglec F, as well as other Siglecs, including Siglec E, which has a high affinity for α2,3-sialylated molecules, on the DCs, may contribute to our observed in vitro and in vivo data. Recently, the role of Siglec E in allorecognition has been elucidated. This receptor plays a key role in inhibiting DC maturation in the presence of DAMPs, which limits the ability of these cells to drive alloreactive T cell proliferation and activation [34]. This needs to be tested further, especially as our previous studies have shown a role for Siglec E in sialyated antigen induced DC tolerance, using Siglec E^−/−^ mice [36].

Our findings highlight that targeting Siglecs on cDC1 and cDC2s using α2,3 Sia-alloantigen may lead to different outcomes for alloreactive T cells. Targeting a sialylated allopeptide to steady-state Batf3-independent cDC2 (CD11b^+^ DCs) led to the deletion/inhibition of indirect allospecific CD4^+^ T cells, in part via Siglec E shown to be expressed on cDC2. These findings agree with our previous publication showing antigen-specific T cell deletion and reduced alloantibodies following targeting DCIR2^+^ on endogenous murine cDC2 (CD8α^−^ DCs) with an MHC I peptide conjugated to a 33D1-crosslinking antibody [27]. Despite this, targeting K^d^ peptide to cDC2s via Siglec engagement did not lead to skin transplant survival, and this outcome complements what we had seen previously when K^d^ peptide was targeted to these cells via the DCIR2^+^ receptor. However, we have yet to confirm the exact mechanism behind the reduction of alloreactive T cells nor have we assessed whether the remaining cells are anergic.

In contrast, interaction with steady state cDC1s presenting alloantigen acquired via Siglec targeting may lead to increased CD62L^+^Foxp3^+^ Tregs, following transplantation, which may contribute to the transplant survival observed. Receptors expressed on resident CD8α^+^ cDC1 and in LNs and XCR1^+^CD8α^+^DEC205^+^ in SPLN or migratory cDC1s (CD103^+^) have been targeted with antigen in vivo to promote antigen-specific T cell tolerance via Treg induction [28,49]. Indeed, Idoyaga et al. (2013) elegantly showed that targeting migratory skin and lung Langerin^+^ CD103^+^ DCs rather than lymphoid resident cDCs was required for Treg induction/expansion [49]. Here we confirmed that targeting antigens to iCD103^+^ BMDCs, with properties aligned to the migratory CD103^+^ DCs, including high levels of LNs homing receptor CCR7 after maturation, via Siglecs led to Treg induction [44].

Several limitations of our study should also be highlighted. The exact mechanism(s) behind graft survival following the targeting of Siglecs in vivo is still to be fully elucidated, as is whether the use of costimulatory blockage or drugs such as Rapamycin yields enhancements with this targeting regime. Although our data suggests that CD4^+^Foxp3^+^CD62L^+^ T cells are increased in a2,3 Sia-K^d^ B6 transplanted mice, we have not tested the antigen specificity capacity of these cells. Lastly, in vitro CD103^+^ targeting was only performed twice. Although in both experiments, we observed that α2,3 Sia-Kd pulsed CD103^+^ cells did not activate TCR75 T cells, caution should be applied when interpreting the data.

The current study provides an insight into the possibility of targeting sialylated alloantigens to Siglec-expressing recipient DCs to promote allograft survival. However, the role of Siglecs on other myeloid cells still requires further investigation. Given our findings in mice, targeting Siglecs in humans to promote allograft survival may be advantageous given their expression on human DCs. Recently, Li et al. (2021) [50] and Lubbers et al. (2021) [51] showed that incubating human monocyte-derived human DCs with α2,3 Sia conjugated to a dendrimeric core led to tolerogenic DCs, capable of promoting Treg induction and/or expansion of natural Tregs, suggesting that this methodology is translatable to a human transplant setting. The findings highlighted here inform on a novel therapeutic strategy to help in preventing graft rejection without the use of prolonged immunosuppressive therapy.

## 4. Materials and Methods

### 4.1. Mice

Female C57BL/6J (B6, H-2^b^) mice (aged 6–8 weeks) were purchased from Charles River Laboratories (Margate, UK). B6.Batf3^−/−^ mice: lacking exons 1 and 2 of the basic leucine zipper transcription factor, ATF-like 3 gene (Batf3) [43], were a kind gift from Dr Kenneth Murphy (Washington University School of Medicine). B6-Tg(TcrαTcrβ)TCR75Rpb mice, which are Rag^−/−^ and CD90.1^+^ (TCR75 Rag^−/−^) [38] and B6.K^d^ mice, B6 expressing a transgene encoding BALB/c MHC I (H-2K^d^), were generated and gifted by Dr Pat Bucy (University of Alabama, Tuscaloosa, AL, USA), and they have been described previously [38]_._ CD4^+^ T cells from the TCR75 mice have indirect specificity for K^d^_54–68_ peptide presented by I-A^b^. The mice were bred and housed at the Biological Services Unit, King’s College London (KCL), under specific pathogen-free conditions. The mice were randomly selected for control and experimental groups. All procedures involving mice were carried out in accordance with the institutional and Home Office Animals Scientific Procedures Act (1986) under the Home Office Project Licence: PPL70/7302 and with institutional approval from King’s College London.

### 4.2. Peptide Conjugates

The K^d^_54–68_ peptide: (QEGPEYWEEQTQRAK), an immunodominant epitope of the α1-chain of the class 1 molecule K^d^, was α2,3 and α2,6 siaylated using our recently published protocol [36]. In addition, peptides were conjugated with FAM5-6 as previously described [36].

### 4.3. Flow Cytometry

For the characterisation of the different APCs and T cells, as well as the evaluation of the Siglec expression, the following fluorochrome-conjugated monoclonal antibodies purchased from ThermoFisher Scientific (Paisley, UK) were used, unless otherwise specified: CD11c APC (clone N418,), B220 APC (RA3 6B2) CD22 PE (Cy34.1, Miltenyi Biotech, Bergisch Gladback, Germany), Siglec H PE (clone 551.3D3, Miltenyi Biotech), CD169 PE (clone RAE197, Miltenyi Biotec) Siglec G PE (clone SH2.1, Miltenyi Biotech), Siglec F PE (clone ES22-10D8, Miltenyi Biotech), Siglec E FITC (clone 8D2, Miltenyi Biotech). MHC Class II I-E/A^b^ FITC (AF6-120.1), MHC Class 1 FITC (clone 28-14-8), CD80 FITC (16-10A1), CD86 FITC (GL1), and CD103 APC (2E7). For T cells the following antibodies were used: CD90.1 PE (clone HIS51,), CD4 PE or FITC (clone RM4-5), CD62L PE (clone MEL-14), and Foxp3 APC (clones FJK-16s).

For flow cytometry analysis, 2 × 10^5^ cells in 100 μLs of PBS containing 2% FCS and 2mM EDTA (FACs Buffer) were incubated for 20 min at 4 °C with anti-CD16/CD32 antibody (clone 93) in 96-well U-bottomed plates before being stained with the appropriate fluorochrome-conjugated antibodies using the manufacturer’s recommendations for 30 min (4 °C). Labelled cells were then washed twice with FACs Buffer. A fluorescence minus 1 control were prepared for each cell marker and used for gating. Stained samples were analysed using either an LSR Fortessa^TM^, BD FACSCelesta^TM^, or BD Accuri C6^TM^ flow cytometer (BD Biosciences, Franklin Lakes, NJ, USA). Acquired data was analysed using FlowJo (version 10.6.1) (FlowJo LLC, Ashland, OR, USA) or BD Accuri C6 software (v 1.0) (BD Biosciences, Franklin Lakes, NJ, USA).

### 4.4. Preparation of Mouse Bone Marrow (BM) DCs (BMDCs) and iCD103 DCs

GM-CSF expanded BMDCs were prepared according to the protocol by Smyth et al. (2013) [15]. Briefly, erythrocytes were lysed using RBC lysis solution (ThermoFisher Scientific), and RBC depleted bone marrow (BM) cells were incubated with the following hydridoma cultures: YTS 191 (anti-CD4; American Type Culture Collection (ATCC), Manassas, VA, USA), YTS 169 (anti-CD8, ATCC), M5/114 (anti-Class II. ATCC), and RA3-3A1 (anti-B220, ATCC). The incubation was performed for 30 min at 4 °C before the cultures were washed and incubated with polyclonal sheep anti-rat IgG Dynabeads^®^ (ThermoFisher Scientific). DC progenitors were isolated by negative selection using a DynaMag^TM^-15 magnet (ThermoFisher Scientific), before being cultured in complete media (RPMI 1640 medium (ThermoFisher Scientific) supplemented with 100 IU/mL penicillin, 100 μg/mL streptomycin, 2 mM L-glutamine, 0.01 M HEPES, 50 mM β2-mercaptoethanol (all from ThermoFisher Scientific), 10% heat-inactivated FCS (PAA, Biopath stores, Cambridge, UK) supplemented with 4 ng/mL GM-CSF for 7 days at 37 °C in 5% CO_2_. The media were replaced on days 2 and 4 of culture. The purity of the DCs was assessed by CD11c^+^ antibody staining and flow cytometry (>80%).

GM-CSF and FLT3L (RD Systems, Abingdon, UK) expanded iCD103 DCs were prepared according to Mayer et al. (2014) [44]. Briefly, 15 × 10^6^ B6 BM cells were expanded in 10mls of complete media supplemented with 200 ng/mL recombinant murine FLT3L (Thermofisher Scientific) and 4 ng/mL GM-CSF for 9 days; non-adherent cells were harvested, counted, and replated at 3 × 10^6^ cells in 10mls of complete media containing the aforementioned growth factors, and the iCD103 cells were harvested on day 16 of culture. CD8α DCs were expanded from B6 BM progenitors using 50 ng/mL FLT3L with a media change on day 5 and cells harvested on day 8 following the protocol of Naik et al. (2005) [45]. iCD103 DC subsets were analysed via flow cytometry for the expression of CD11c and CD103. CD8α^+^ DC subsets were analysed for expression of CD11c, CD11b, B220, and CD24.

### 4.5. Preparation of DCs from Mouse Spleen and Lymph Nodes

Spleens isolated from either B6 or B6.Batf3^−/−^ mice were diced into small sections using a Swann–Morton sterile blade (Appleton Wood, Birmingham, UK), and a single cell suspension was made by digesting the aforementioned in PBS supplemented with 0.5% collagenase (Merck, Dorset, UK) and 10 µM/mL DNAase (Merck) for 30 min at 37 °C. RBC-free splenocytes were passed through a 70 µm cell strainer (Merck) to obtain a single-cell suspension before DCs were incubated with anti-CD11c microbeads and isolated using MS/LS columns and an OctoMACs magnet (Miltenyi Biotech) according to the manufacturer’s protocol.

### 4.6. In Vitro Peptide-DC Binding

A total of 0.5–1 × 10^6^ B6 and B6.Batf3^−/−^ derived BMDCs, iCD103^+^DCs, or SPLN-DCs, in 100 μL of complete culture media, were incubated with 10 μg/mL of either K^d^, α2,3 Sia-K^d^, or α2,6 Sia-K^d^ FAM5/6 peptides for 4 h at 37 °C before excess peptide was removed by washing with RPMI. The cells were counted, and 0.5 × 10^6^ cells were stained with APC conjugated antibodies to CD11c (DCs) or CD103 (iCD103^+^ DC). The cells were assessed using flow cytometry.

### 4.7. CD4^+^ T Cell Proliferation Assays

Responder CD4^+^ T cells were isolated from TCR75 Rag^−/−^ mice. A single cell suspension was obtained by passing the spleens through a 70 um cell strainer (Fisher Scientific, Loughborough, UK), and erythrocytes were lysed using RBC lysis solution (ThermoFisher Scientific). The remaining T cells were labelled with 1 µM Vybrant™ CFDA SE (CFSE (5) and-6)-Carboxyfluorescein Diacetate, Succinimidyl Ester), following the manufacturer’s protocols (ThermoFisher Scientific). B6 BMDCs, B cells, and iCD103 DCs were pulsed with 10 μg/mL of either K^d^ or α2,3 Sia-K^d^ or α2,6 Sia-K^d^ peptides for 4 h at 37 °C before being co-cultured with CFSE labelled TCR75 CD4^+^ T cells at a ratio of 1:10 DC:T cell or 1:10 B:T cell for 3 days. Non-pulsed DCs served as controls. Proliferation of TCR75 CD4^+^ was measured as the CFSE dilution on days specified using flow cytometry. The gating strategy is shown in Supplementary Figure S5.

### 4.8. Cytokine Specific ELISAs

To measure IL-2 and IFN-γ, culture supernatants taken from the above cultures were measured using an IL-2- or IFN-γ-specific sandwich enzyme-linked immunosorbent assay (ELISA) kit, following the manufacturer’s instructions (ThermoFisher Scientific). All supernatants were diluted at 1:10 with an ELISA diluent before use. Each sample was tested as a technical replicate, and the mean (pg/mL) +/− SEM for multiple experiments is shown. Absorbance was read using a Multi-Mode Reader (Synergy HTX, BioTek, Winooski, VT, USA).

### 4.9. In Vitro Treg Induction Assay

B6 BMDCs and iCD103 DCs were treated with either 10 μg/mL α2,3 Sia-K^d^, α2,6 Sia-K^d^, or K^d^ as previously mentioned. CFSE labelled TCR75 CD4^+^ T cells were co-cultured with the aforementioned APCs at a 1:10 ratio in the presence of 5U/mL recombinant human IL-2 (Proleukin-Novrtis, Surrey, UK) in a 96 U-bottomed plate at 37 °C/5%CO_2_ for 3 days. For intracellular staining, with an anti-Foxp3 APC antibody (clone FJK-16s, Thermofisher Scientific), the cells were fixed and permeabilised using a Foxp3/Transcription Factor Staining Buffer kit according to the manufacturer’s protocol (ThermoFisher Scientific). The cells were stained with anti-CD4^+^ PE labelled antibody Foxp3 expression on CD4^+^ T cells, assessed by flow cytometry analysis.

### 4.10. Skin Transplantation

A total of 10 μg (in 200 μL saline) of K^d^, α2,3 Sia-K^d^, or α2,6 Sia-K^d^ peptides was administered intravenously (i.v) to either B6, B6Rag^−/−^, or B6.Batf3^−/−^ recipient mice 1 day (day-1) prior to receiving either a B6.K^d^ or BALB/c skin transplant. Skin transplants were performed as previously described [15]. In brief, mice were anesthetized using IsoFlo isoflurane (Zoetis, Kalamazoo, MI, USA), and a full-thickness B6.K^d^ donor tail skin was mounted onto the dorsal thorax of recipient mice via suturing using 45 mm polyamide sutures, 18 (Ethilon, Cornelia, GA, USA), and secured with a waterproof Elastoplast plaster for 7 days. Grafts were observed daily, and rejection was considered as greater than 90% necrosis of donor tissue, assessed by visual inspection. B6 recipient mice received 250 μg of anti-CD8 antibody (YTS169, ATCC) in 100 μL of saline via intraperitoneal (ip) to depleted CD8^+^ cells one day prior and after skin transplant and weekly post-transplant date. Graft survival between groups was compared using the log-rank test.

### 4.11. Treg Analysis in Transplant Recipients

Recipient mice were bled from the tail vein using a 26^1/2^ G needle into a Microvette CB 300 tube lined with EDTA (Sarstedt, Numbrecht, Germany) 14 days post transplantation. RBC-free cells were stained with fluorescently labelled anti-CD4 and CD62L antibodies and subsequently intracellularly stained for Foxp3 using an anti-FoxP3 antibodies/kit following the manufacturer’s protocol (ThermoFisher Scientific). Foxp3 and CD62L expression on CD4^+^ T cells was assessed by flow cytometry analysis.

### 4.12. CD4^+^ T Cell Adoptive Transfer

T cells were isolated from the spleens of TCR75 Rag^−/−^ mice as described above. Recipient mice received 2 × 10^6^ TCR75 CD4^+^ T cells (CD90.1^+^)) via i.v. injection, and 24 h later, the mice were injected i.v with either 10 μg/mL of α2,3 Sia-K^d^, α2,6 Sia-K^d^, or K^d^ peptide. Ten days later, the mice were culled, and their lymph nodes (LN) and spleens (SPLN) were removed. RBC-free single cells were isolated from these tissues as described and stained with fluorescently labelled anti-CD90.1 and anti-CD4 antibodies, before being assessed via flow cytometry.

For adoptive transfer to B6.Rag^−/−^ recipients, CD4^+^ T cells were isolated from B6 spleens using a CD4^+^ Untouched Isolation Kit (Thermofisher Scientific), following the manufacturer’s instructions. Recipient B6.Rag^−/−^ mice received 0.5 × 10^6^ B6 CD4^+^ cells in 200 μLs saline via i.v injection one day before skin transplantation.

### 4.13. Statistical Analysis

Statistical analysis was performed using an unpaired Student’s *t* test for the measurement of two data sets; one-way ANOVA with Tukey’s multiple comparisons test was used for the measurement of two or more data sets with one independent variable using GraphPad Prism (version 10.3.1) (GraphPad Software, La Jolla, CA, USA). The median survival time (MST) of the skin grafts was calculated using Mantel Cox and log-rank test using GraphPad Prism. The data shown is mean ± standard error of the mean (SEM).

## Data Availability

The data sets generated during and/or analysed during the current study are available from the corresponding author on reasonable request.

## Supplementary Materials

The following supporting information can be downloaded at: https://www.mdpi.com/article/10.3390/ijms26136168/s1.
