# Supplementary material for: Targeting Recipient Dendritic Cells with Sialic Acid-Modified Donor Alloantigen Prolongs Skin Transplant Survival
Source: Int J Mol Sci. 2025 Jun 26;26(13):6168. doi: 10.3390/ijms26136168 (PMC12250551; doi:10.3390/ijms26136168)
Supplement: Supplementary file 1 [file ijms-26-06168-s001.zip › ijms-3644402-supplementary.pdf]

Supplementary Materials

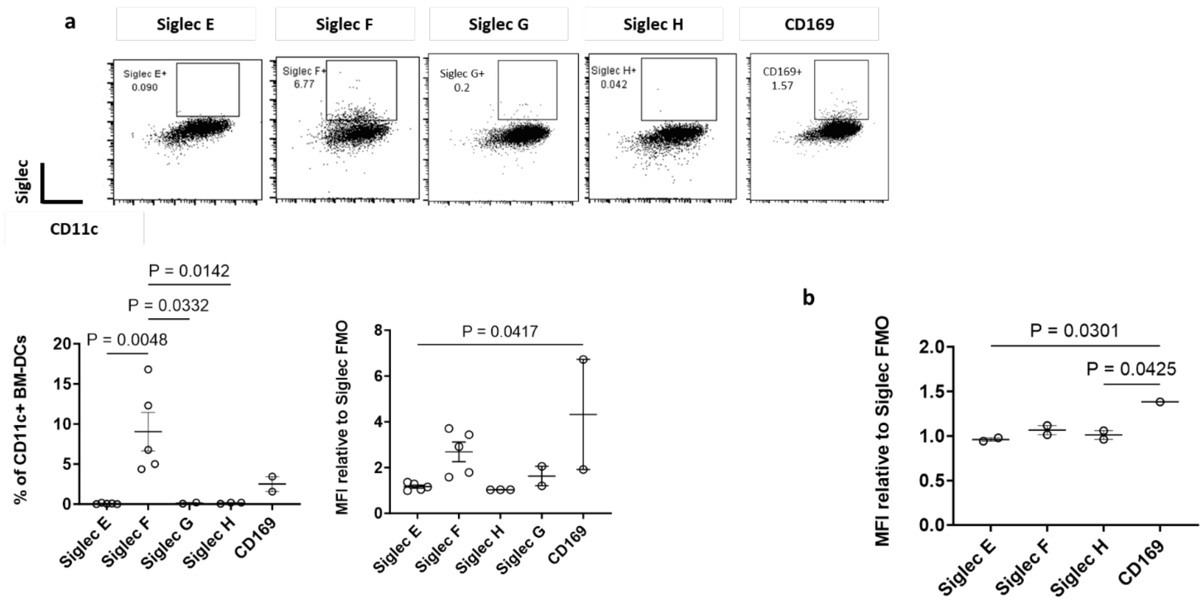

Supplementary Figure S 1. Siglec-expression on BMDC and SPLN CD11c+ DCs. (a). B6 BMDCs and (b). CD11c+ splenic DCs isolated by positive bead selection were stained with anti-mouse CD11c APC/PE and either anti-mouse Siglec E- FITC, Siglec F- PE, Siglec G- APC, Siglec H-PE and CD169-PE. Live cells were gated on FSC, SSC, followed by doublet exclusion and expression of each Sigle was analysed on CD11c+ cells. Plots represent the MFI of Siglec-expression DCs and represents data from 3 independent experiments. Statistical comparison was performed using One-way ANOVA and Tukey's multiple comparisons test.

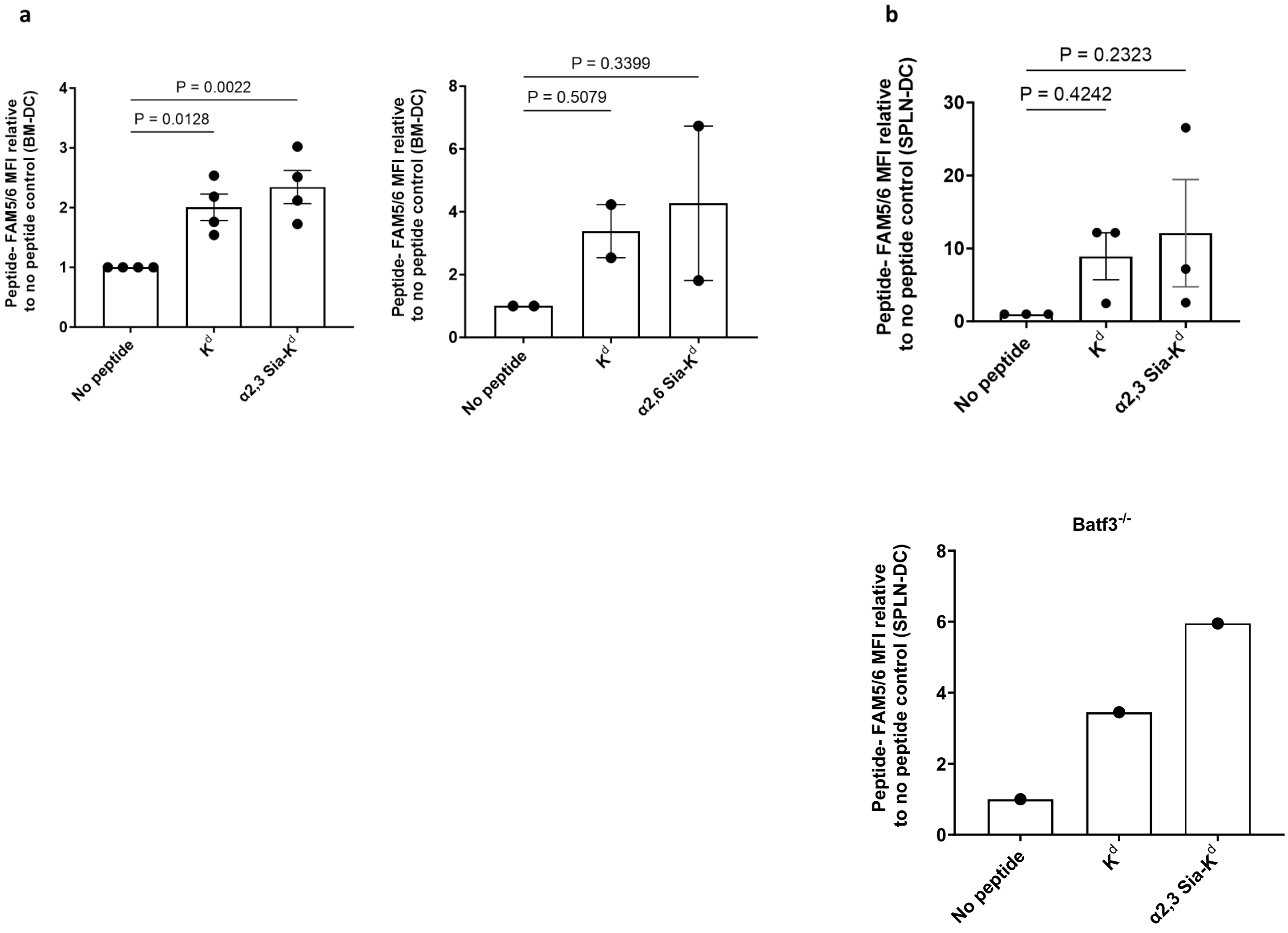

Supplemental Figure S2. FAM 5 conjugated α2,3 Sia-K^d and α2,6 Sia-K^d complexes bind to DC *in vitro*. (a) B6 BMDCs and (b) B6 and Batf3$^{-/-}$ SPLN DCs were pulsed with either 10 µg/ml K^d or α2,3 Sia-K^d or α2,6 Sia-K^d – FAM5 labelled peptide for 4 hours. Live cells were gated on FSC, SSC, followed by doublet exclusion and presence of FITC staining was analysed on CD11c$^+$ cells using flow cytometry. Unpulsed cells were the negative controls (no peptide). The fold increase in FITC expression compared to the no peptide control (set to a value of 1) is shown and each bar presents the Mean +/- SEM from four independent experiments. Peptide binding to SPLN-DCs, data presented was pooled from 3 or 1 independent experiments (B6 and Batf3-/- mice, respectively). Statistical comparison was performed using One-way ANOVA and Tukey’s multiple comparisons test.

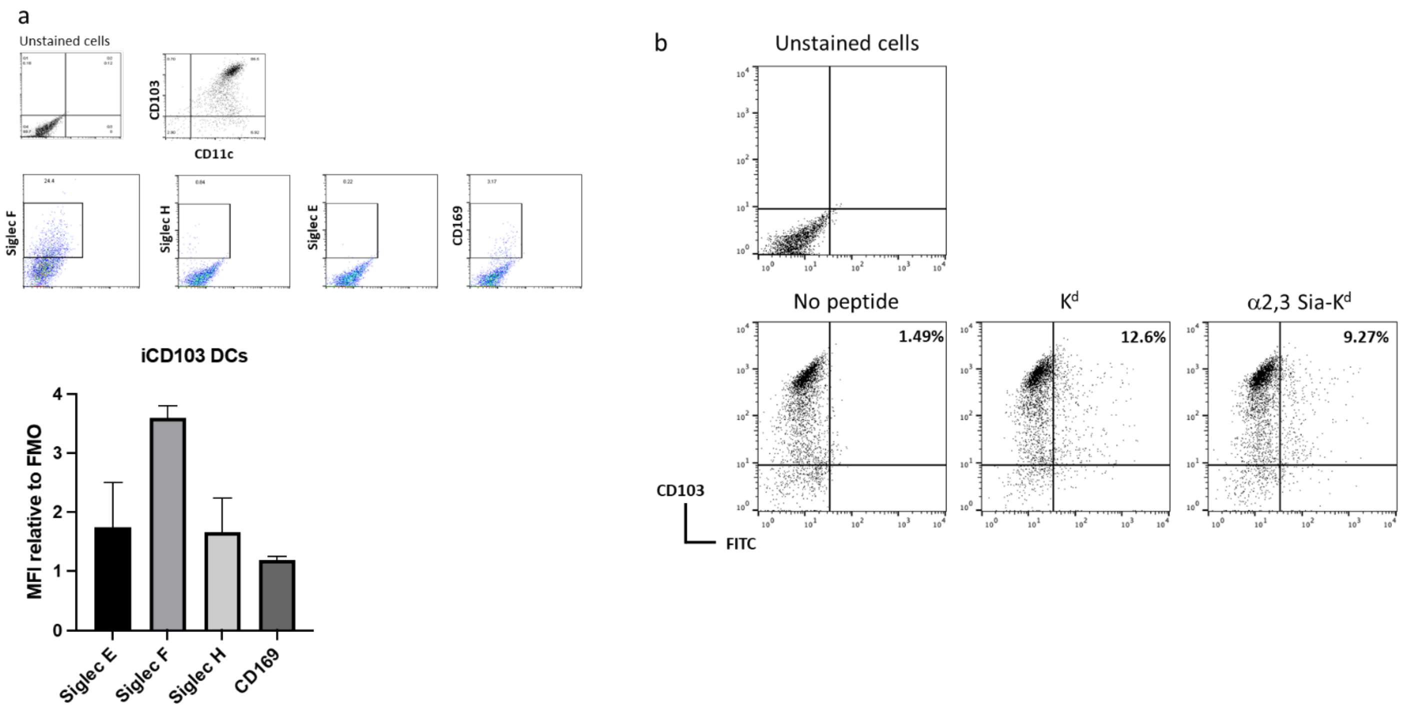

Supplemental Figure. S3. Siglec-expression on iCD103 DCs and acquisition of α2,3 Sia-K^d- FITC. (A). iCD103$^+$ BMDC were stained with anti-mouse CD11c APC/PE and either anti-mouse Siglec E- FITC, Siglec F- PE, Siglec G- APC, Siglec H-PE and CD169-PE. Plots represent the MFI of each Siglec expression on CD11c$^+$ cells and represents data from 2 independent experiments. (B). iCD103$^+$ BMDC were pulsed with either 10 µg/ml K^d or α2,3 Sia-K^d- FAM5 labelled peptide. Unpulsed cells were the negative controls. Live cells were gated on FSC, SSC, followed by doublet exclusion and presence of staining was analysed on CD103$^+$ cells using flow cytometry. Data presents 3 independent experiments.

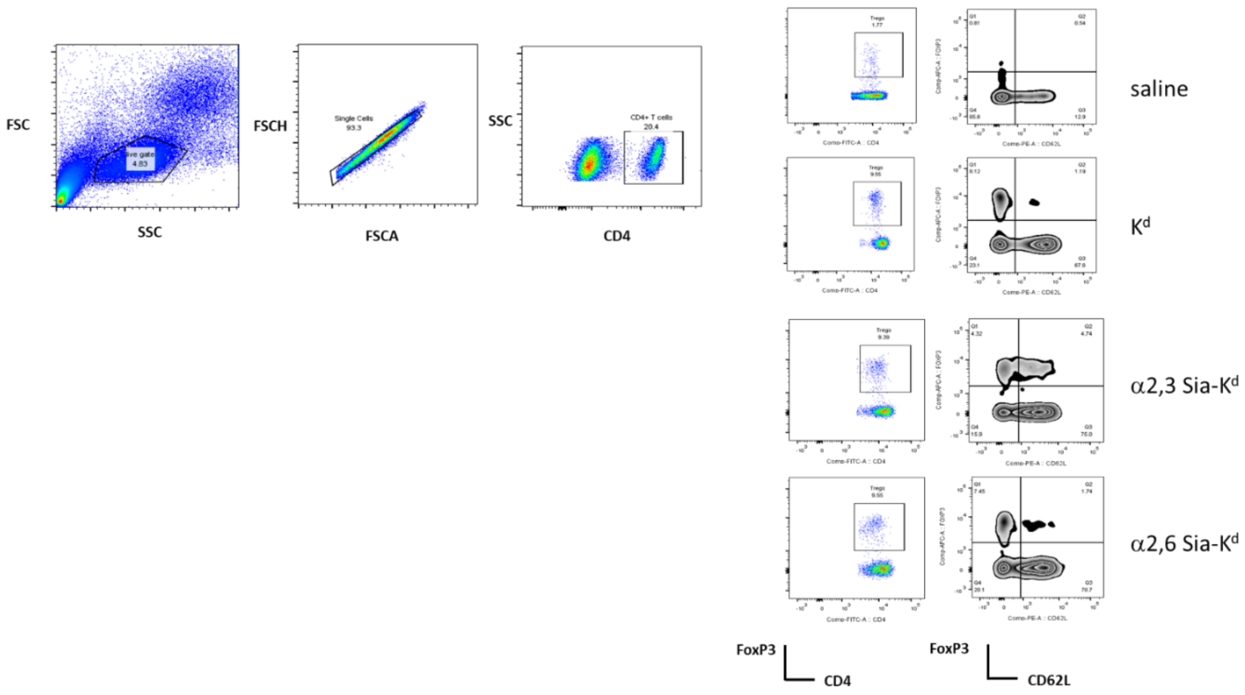

Supplemental Figure. S4. Gating strategy to assess FoxP3$^+$ and CD62L$^+$ expression. Live cells were gated on FSC, SSC, followed by doublet exclusion and presence of CD4$^+$ cells analysed. Expression of Foxp3 and CD62L in CD4$^+$ cells is shown.

a

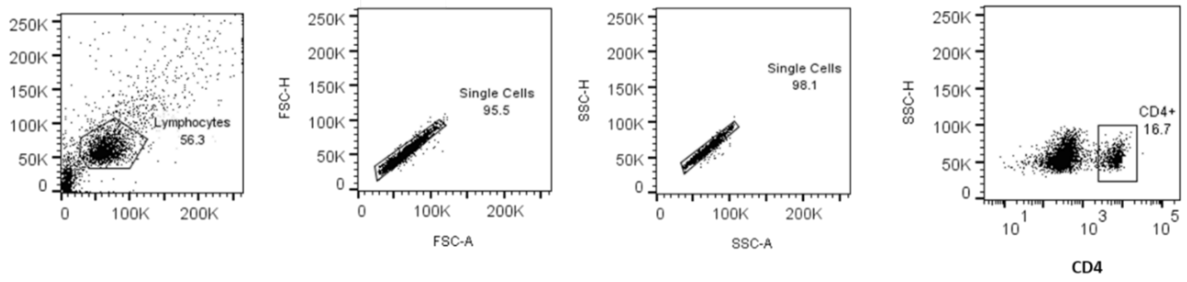

Supplemental Figure. S5. Gating strategy to assess presence of CD4^{+} T cells, T cell activation and Foxp3 expression**.** Live cells were gated on FSC, SSC, followed by doublet exclusion and presence of CD4^{+} cells analysed.
